# Long-term outcomes of Aripiprazole long-acting injectable: a 10-year mirror image study of patient acceptability and treatment effectiveness

**DOI:** 10.1038/s41537-025-00637-7

**Published:** 2025-06-23

**Authors:** Joshua Barnett, Sofia Pappa

**Affiliations:** 1 West London NHS Trust, London, UK; 2 East London NHS Foundation Trust, London, UK; 3 Department of Brain Sciences, Faculty of Medicine, Imperial College, London, UK

**Keywords:** Psychosis, Schizophrenia

## Abstract

Relapses are frequent in schizophrenia and other psychotic disorders. While long-acting injectable antipsychotics (LAIs) are effective in preventing hospital admissions and improving adherence and patient outcomes, they are still under-utilised. Furthermore, evidence from newer formulations and longitudinal studies, despite their commonly long-term use, remains limited. To address this scarcity of data, this study aims to evaluate the long-term effectiveness and acceptability of once-monthly Aripiprazole long-acting injectable (ALAI), the only third-generation antipsychotic available in long-acting formulation. In this pragmatic, independent, ten-year mirror-image study conducted within a large urban mental health service in London, UK, we assessed hospital admission rates and treatment retention over 5 years following ALAI initiation in a naturalistic adult cohort. Frequency and length of hospitalisations in the 5 years pre- and post-initiation were recorded using electronic records, as were discontinuation rates and reasons. Separate analyses were performed comparing outcomes between treatment completers and discontinuers, as well as between those with schizophrenia vs other diagnoses. In total, 135 patients were included in the study (63% with Schizophrenia, 37% with other diagnoses). The discontinuation rate was 47% at 5 years (23.7%, 13.6%, 7.9%, 7.3% and 5.3% in years 1 to 5 respectively). Among the 53% who completed 5 years of ALAI treatment, we observed an 88.5% reduction in mean number (1.57 to 0.18, *p* < 0.001) and a 90% reduction in mean length of hospitalizations compared to 5 years pre-ALAI initiation (103 to 10 days, *p* < 0.0001). Median admissions and length fell from 1 to 0 and 68 to 0 days (*p* < 0.001), respectively. In contrast, discontinuers (47%) exhibited inferior outcomes and showed only a 29.9% reduction in admissions over 5 years. Patients were more likely to discontinue due to poor compliance and ineffectiveness and rarely due to tolerability issues. Apart from switching to ALAI from another LAI, there were no major clinical or demographic predictors of treatment continuation. Outcomes were consistent independent of diagnosis. Potential confounders however must not be overlooked, such as the exclusion of a large number of patients due to strict eligibility criteria as well as changes to healthcare policy over the study period. This is the first study to report 5-year hospitalisation and treatment persistence outcomes with ALAI. Its sustained use was associated with substantial reductions in hospital use, with 85% of completers requiring no further admissions, compared to 30% of discontinuers. These real-world findings support the long-term value of ALAI and may help address common barriers to LAI adoption in clinical decision-making.

## Introduction

Schizophrenia is a chronic and severe mental disorder^1^ characterised by multiple relapses during the course of the illness^2^ which are often inevitable if left untreated^3^. It is associated with significant morbidity that often negatively affects educational and occupational output^4^ and contributes to increased economic strain on healthcare systems^5,6^.

Antipsychotic medications serve as the foundation of pharmacological treatment for Schizophrenia and other psychotic disorders^7^. They are effective in alleviating symptoms^8^, reducing the risk of relapse^9^ and enhancing overall functionality^10^. Non-adherence to medication is however prevalent and is closely linked to a heightened risk of relapse and hospitalisation^11^, functional decline^12^ and increased mortality^13^. The introduction of long-acting injectable (LAI) antipsychotics have helped to address these challenges by providing a safe and effective^14^ method of improving treatment adherence^14–16^ that have also been shown to reduce relapse rates^17^ and healthcare costs^18^.

However, there is a relative scarcity of data on the real-world effectiveness of newer LAIs and particularly of Aripiprazole long-acting injectable (ALAI) over prolonged periods of time. ALAI is a newer atypical LAI that has been commonly prescribed and has gained popularity over the last decade^19^. Furthermore, it is the only partial agonist available in long-acting formulation and despite its higher acquisition cost compared to first generation antipsychotics^20^, it is often associated with a more favourable side effect profile^21^ and improved patient outcomes^22^.

A number of naturalistic studies have demonstrated the positive effects of other second-generation LAI antipsychotics^4,23^, underscoring their significance in the practical management of schizophrenia and other psychotic disorders. This is particularly crucial given the inherent challenges in treating these conditions, including complex symptom management and the need for individualized care strategies^24^. However, while existing studies have demonstrated short- to medium-term benefits, most have focused on follow-up periods of 12 months or less, often in controlled or trial settings that may not reflect routine clinical practice^25,26^. In addition, few studies have explored patient outcomes such as relapse and treatment acceptability and perseverance rather than symptoms control over extended timeframes, which is particularly important given their frequent use as ongoing, and often lifelong, maintenance treatment in schizophrenia.

The primary objective of this study was, therefore, to evaluate the long-term clinical outcomes associated with once-monthly ALAI, specifically focusing on hospitalisation rates and treatment discontinuation. We hypothesized that ALAI would significantly reduce hospitalisations over five years, reflecting its potential for sustained efficacy and improved adherence. By examining these outcomes, the study sought to provide insights into the real-world, long-term impact of ALAI use in clinical practice.

## Results

A total of 210 patients were registered for treatment with ALAI during the study period. Of these, 32 were initiated on ALAI in a forensic setting, 26 had incomplete data available for various reasons and 17 passed away during the study period (none of these deaths were deemed to be causally related or directly linked to any concomitant or previous use of ALAI).

The remaining 135 patients all met eligibility criteria and were included in the study: 85 patients (63%) had a primary diagnosis of schizophrenia and the other 50 (37%) had schizoaffective disorder, bipolar affective disorder or another diagnosis. Of the total cohort, 57% were male, 38.5% were white, while 21% carried a formal diagnosis of comorbid substance use disorder. The mean age of the group was 51.6 years with a mean number of total previous admission of 3.9. 56% started treatment with ALAI in an inpatient and 44% in an outpatient setting with the majority switching to ALAI from an oral antipsychotic (67%). The main reasons for switching to ALAI were poor compliance (63%) and tolerability issues (27%). The demographic and clinical characteristics of the total cohort and the schizophrenia-only group are summarized in Table 1.

**Table 1:** Demographical and clinical characteristics of total patient cohort.

Characteristics	All patients *n* = 135 (%)	Schizophrenia *n* = 85 (%)	Other diagnoses *n* = 50 (%)
Gender			
Male	77 (57)	52 (61.2)	25 (50)
Female	58 (43)	33 (38.8)	25 (50)
Age			
Mean (SD), range	51.64 (15.53), 25–92	51.14 (15.83), 25–88	52.48 (15.13), 25–92
Ethnicity			
White	52 (38.5)	30 (35.3)	22 (44)
Non-white	83 (61.5)	55 (64.7)	28 (56)
Primary diagnosis			
Schizophrenia	85 (63)	85 (100)	
Schizoaffective disorder	13 (9.6)		13 (26)
Bipolar affective disorder	20 (14.8)		20 (40)
Other	17 (12.6)		17 (34)
Comorbid substance misuse			
Yes	28 (20.7)	19 (22.4)	9 (18)
No	107 (79.3)	66 (77.6)	41 (82)
Care Setting			
Inpatient	75 (55.6)	45 (52.9)	30 (60)
Outpatient	60 (44.4)	40 (47.1)	20 (40)
No. previous admissions			
Mean (SD), range	3.97 (4.48), 0–24	3.54 (3.93), 0–22	4.7 (5.23), 0–24
Antipsychotic switched from			
None	2 (1.5)	0 (0)	2 (4)
Oral Aripiprazole	57 (42.2)	31 (36.5)	26 (52)
Oral other	33 (24.4)	23 (27.1)	10 (20)
Depot/LAI Other	43 (31.9)	31 (36.5)	12 (24)
Reason for switching from previous medication			
Poor compliance	85 (63)	51 (60)	34 (68)
Poor tolerability	37 (27.4)	26 (30.6)	11 (22)
Ineffectiveness	5 (3.7)	4 (4.7)	1 (2)
Patient preference/choice	2 (1.5)	0 (0)	2 (2)
Other	4 (3)	4 (4.7)	0 (0)

### Discontinuation rates and reasons

In total, 63/135 (46.7%) discontinued and 72/135 (53.3%) patients of the total cohort continued treatment at 5 years. Similarly, 40/85 (47.1%) stopped and 45/85 (52.9%) patients in the schizophrenia-only group completed 5 years of treatment with ALAI; this information is captured in Table 2.

**Table 2:** Continuation rates for all patients and schizophrenia patients.

Diagnosis	Year 1	Year 2	Year 3	Year 4	Year 5	
All patients	76.3% (103/135)	65.9% (89/135)	60.7% (82/135)	56.3% (76/135)		53.3% (72/135)
Schizophrenia	76.5% (65/85)	64.7% (55/85)	58.8% (50/85)	55.3% (47/85)		52.9% (45/85)

In the all-patient group, 23.7% (32/135) discontinued in the first year, 13.6% (14/103) in second, 7.9% (7/89) in the third, 7.3% (6/82) the fourth and 5.3% (4/76) in year 5. Hence, 76.3% of patients continued ALAI for 1 year, 65.9% for 2 years, 60.7% for 3 years, 56.3% for 4 years and 53.3% for 5 years. In the Schizophrenia group, 23.5% (20/85) discontinued in year 1, 15.4% (10/65) in year 2, 9.1% (5/55) in year 3, 6% (3/50) in year 4 and 4.3% (2/47) in year 5; the continuation rates were 76.5% in the first year, 64.7% in the second, 58.8% in the third, 55.3% the fourth and 52.9% for the full 5 years.

The main reasons for discontinuation (Table 3) included poor compliance (24.4%) and ineffectiveness of the medication (14.1%). Poor tolerability was not a common reason for discontinuation, with only 6.9% of patients (4.7% (4/85) with Schizophrenia and 10% (5/50) with other diagnoses) reporting this as an issue. A third (3/9) of those who discontinued due to poor tolerability reported experiencing akathisia, while 22.2% (2/9) reported weight gain. Postural hypotension, sedation, ECG changes and other side effects were all reported once each.

**Table 3:** Discontinuation reasons for all patients, schizophrenia patients and patients with other diagnoses.

Poor Compliance	33 (24.4)	22 (25.9)	11 (22)
Ineffectiveness	19 (14.1)	13 (15.3)	6 (12)
Poor Tolerability	9 (6.7)	4 (4.7)	5 (10)
Other	2 (1.5)	1 (1.2)	1 (2)

Significant differences in demographic and clinical characteristics between the group who continued ALAI for the full 5 years versus those who didn’t, were calculated using a multivariate logistic analysis, with the results displayed in Table 4. The main predictor of continuation was switching from an alternative depot/LAI medication prior to initiation of ALAI with no other significant differences in demographic or clinical factors discriminating between continues and discontinuers.

**Table 4:** Demographical and clinical characteristics of patients who continued ALAI for 5 years vs patients who discontinued.

Characteristics	Continued ALAI *n* = 72 (%)	Discontinued ALAI *n* = 63 (%)	X 2 (d.f)	*P*-value
Gender				
Male	41 (56.9)	36 (57.1)	0.01 (1)	0.981
Female	31 (43.1)	27 (42.9)		
Age				
Mean (SD), range	54.86 (15.17), 27–92	47.95 (15.23), 25–80		
Ethnicity				
White	25 (34.7)	27 (42.9)	0.939 (1)	0.333
Non-white	47 (65.3)	36 (57.1)		
Primary diagnosis				
Schizophrenia	45 (62.5)	40 (63.5)	0.302 (3)	0.96
Schizoaffective disorder	7 (9.7)	6 (9.5)		
Bipolar affective disorder	10 (13.9)	10 (15.9)		
Other	10 (13.9)	7 (11.1)		
Comorbid substance misuse				
Yes	12 (16.7)	16 (25.4)	1.558 (1)	0.212
No	60 (83.3)	47 (74.6)		
Care Setting				
Inpatient	37 (51.4)	38 (60.3)	1.085 (1)	0.298
Outpatient	35 (48.6)	25 (39.7)		
No. previous Admissions				
Mean (SD), range		4.33 (4.35), 0–19		
Antipsychotic switched from				
Oral Aripiprazole	26 (36.1)	31 (49.2)	2.42 (1)	0.12
Oral/Depot Other	45 (62.5)	31 (49.2)		
Antipsychotic switched from				
None	1 (1.4)	1 (1.6)	5.124 (3)	0.163
Oral Aripiprazole	26 (36.1)	31 (49.2)		
Oral Other	16 (22.2)	17 (27)		
Depot Other	29 (40.3)	14 (22.2)		
Oral	42 (58.3)	48 (76.2)	**5.047 (1)**	**0.025**
Depot/LAI	29 (40.3)	14 (22.2)		
Reasons for switching				
Poor Compliance	39 (54.2)	46 (73)	9.207 (5)	0.101
Poor Tolerability	23 (31.9)	14 (22.2)		
Ineffectiveness	5 (6.9)	0 (0)		
Patient Choice	2 (2.8)	0 (0)		
Other	2 (2.8)	2 (3.2)		

Bold values identify statistical significance (*p* < 0.05).

### Hospitalisation rates

**Fig. 1:**
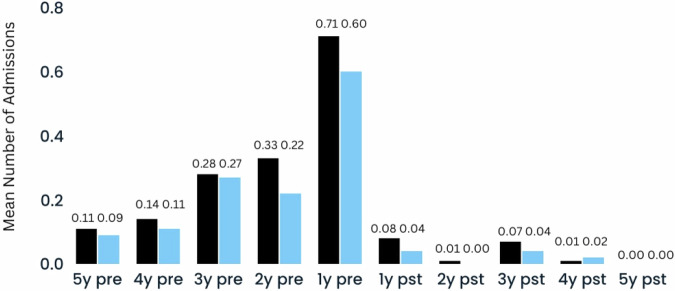
Mean number of admissions per year in all patient group (*n* = 72; black) and schizophrenia group (*n* = 45, blue) in the 5 years pre- and post-ALAI treatment initiation, for those who completed 5 years of treatment (continuers).

**Fig. 2:**
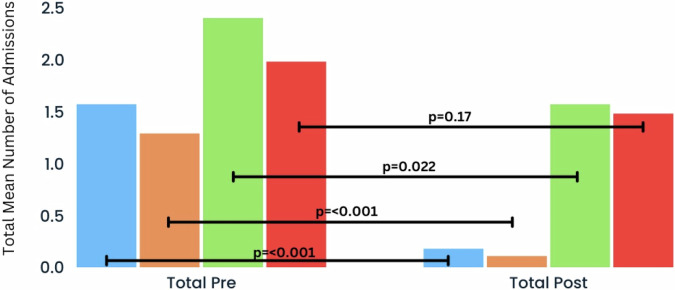
Total mean number of admissions in 5 years pre- and post-ALAI treatment initiation in all patient group (*n* = 72, blue) and schizophrenia group (*n* = 45, orange) who continued (continuers), and the all patient group (*n* = 63, green) and schizophrenia group (*n* = 40, red) who discontinued (discontinuers).

In the patients who continued with ALAI for 5 years (*n* = 72, 53%), the mean [standard deviation (SD)] number of admissions decreased from 1.57 (1.31) per patient in the 5 years prior to ALAI initiation to 0.18 (0.48) in the 5 years after initiation (Figs. 1 & 2). The median number of admissions fell from 1 to 0 (*P* < 0.001, Wilcoxon signed-rank test). Similarly, the mean length of admission fell from 103.06 (124.93) days in the 5 years pre-ALAI initiation to 10.01 (28.74) days in the 5 years post-ALAI initiation (Figs. 1 & 2), while the median length of admission fell from 68 days to 0 days in the same period (*P* < 0.001, Wilcoxon signed-rank test).

**Fig. 3:**
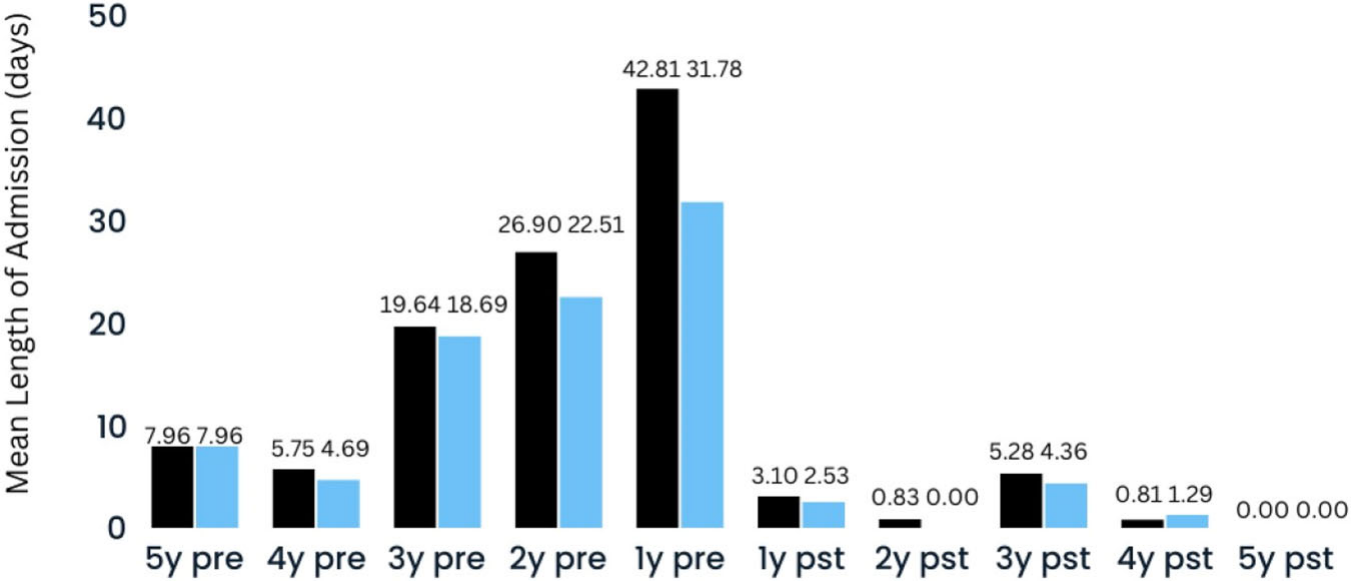
Mean length of admissions (days) per year in all patient group (*n* = 72; black) and schizophrenia group (*n* = 45, blue) in the 5 years pre- and post-ALAI treatment initiation, for those who completed 5 years of treatment (continuers).

**Fig. 4:**
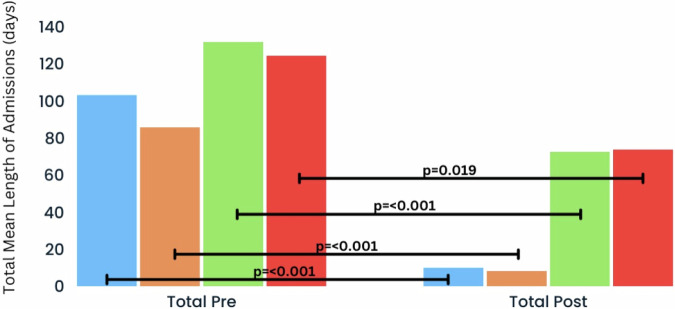
Total mean length of admissions (days) in 5 years pre- and post-ALAI treatment initiation in all patient group (*n* = 72, blue) and schizophrenia group (*n* = 45, orange) who continued (continuers), and the all patient group (*n* = 63, green) and schizophrenia group (*n* = 40, red) who discontinued (discontinuers).

The schizophrenia-only patient group who completed 5 years of ALAI (*n* = 45) demonstrated similar statistically significant reductions in number and length of admissions (Figs. 3 and 4). In the 5 years pre-ALAI initiation to the 5 years post, the mean number of admissions decreased from 1.29 (1.18) to 0.11 (0.38) (*P* < 0.001, Wilcoxon signed-rank test) while the mean length decreased from 85.62 (112.38) to 8.18 (28.54) (*P* < 0.001, Wilcoxon signed-rank test).

In the group of discontinuers (*n* = 63), a significant reduction was seen in the mean (SD) number of admissions from 2.4 (1.9) to 1.57 (1.89) (*P* = 0.022, Wilcoxon signed-rank test) as well as the mean length of admission from 131.62 (124.66) to 72.44 (87.01) (*P* < 0.001, Wilcoxon signed-rank test), though the improvement is much more modest than the continuers group. In the schizophrenia group (*n* = 40), there was a numerical, but statistically insignificant reduction in the number of hospital admissions from 1.98 (1.87) to 1.48 (1.34) (*P* = 0.17, Wilcoxon signed-rank test), along with a statistically significant decrease in length of hospitalisation from 124.25 (131.87) to 73.73 (80.32) (*P* = 0.019, Wilcoxon signed-rank test). Again, the reduction in length of hospital admission is more modest than the schizophrenia group who continued treatment throughout the 5 years.

## Discussion

Our study offers one of the longest follow-up periods for a naturalistic cohort treated with an LAI antipsychotic medication. The use of ALAI was associated with high continuation rates and significantly reduced hospitalisation rates, with these benefits sustained well into the fifth year of treatment. Over half of the patients (53%) maintained ALAI therapy 5 years post-initiation and importantly 85%required no hospital admissions during follow-up. In contrast, those who discontinued the medication during the study period (47%) demonstrated overall less favourable outcomes with only 30% not needing a hospital admission at 5 years follow-up.

### Impact on hospitalisations

This 10-year mirror-image study showed substantial reductions in both frequency and duration of hospital admissions, both of which persisted over the 5-year follow up period (Figs. 1 and 3). Those who continued treatment for 5 years (*n* = 72, 53%) demonstrated an overall reduction of 88.5% in the mean number of admissions and 90.3% in the mean length of admissions compared to the 5-year period before medication initiation while 84.7% (61/72) of patients did not have any admissions during the follow-up period. In contrast to those who completed 5 years, those who discontinued treatment with ALAI (*n* = 63, 47%) exhibited inferior outcomes. The overall mean number and length of admissions fell by only 29.9% and 44.8% respectively, while only 30.2% (19/63) of discontinuers had no admissions in the follow up period.

The trend of reduced hospitalisation can be seen in previous naturalistic studies, though the studies examining ALAI have shorter follow-up periods of 1 year^20,27^ and 2 years^2^ respectively. Overall, the findings from this study demonstrated more favourable outcomes, which were maintained into the third, fourth and fifth year of treatment, with minimal reliance on hospital-based care within this patient cohort. A more direct comparison can be made with a 10-year mirror-image study by Pappa et al.^23^ examining Paliperidone 1-monthly LAI (PP1M) use over the same 5-year follow up period. It showed that continuing PP1M for 5 years also reduced mean number (1.84–0.51) and length (102.3–32.7 days) of hospitalisations when compared to the 5 years pre-initiation. The decrease in mean number is similar to ALAI (1.57–0.18), though it was outperformed in terms of duration (103.06 to 10.01). Thus, patients who continued treatment for 5 years (*n* = 77) demonstrated an overall reduction of 72.3% in the mean number of admissions and 68% in the mean length of admissions compared to the 5-year period before initiation (as opposed to 89% and 90% with ALAI respectively). These findings may suggest that ALAI may be superior in preventing or minimising bed usage, though it worth noting that other studies have shown that clinicians were more likely to prescribe PP1M over ALAI in more symptomatic patients^28^ with greater frequency and length of hospital admissions prior to initiation^29^, hence personalising treatment choice to clinical need.

Furthermore, both the frequency and duration of hospital admissions were on an upward trajectory in the five-year pre-initiation period, peaking in the year immediately preceding ALAI initiation in both the overall cohort and the schizophrenia-only subgroup (Figs. 1 and 3). This finding is consistent with other studies investigating both ALAI^2^ and PP1M as well as other LAIs^23,30^. The observed pattern suggests that LAIs are frequently initiated following extended periods of clinical decline, despite emerging evidence supporting the advantages of early LAI initiation^31^ and that barriers to the implementation of these therapies persist including logistical challenges, negative attitudes towards injectable medication as well as concerns about side effects and cost^32,33^. To this end, it is also noteworthy, that at the time of ALAI initiation, none of the 85 patients with schizophrenia were formally diagnosed with or recorded/coded as treatment-resistant Schizophrenia. As shown in Table 1, 23 patients were switched from a non-aripiprazole oral antipsychotic and 31 patients were switched from a non-aripiprazole LAI antipsychotic. However, only 4 out of the total 85 patients switched to ALA due to ineffectiveness of an alternative antipsychotic, hence minimizing the risk of treatment resistant cases that may have not been pick up as such.

Studies typically lack follow-up on patients who discontinue or drop out of interventions. However, Taylor et al.^30^ (a 4-year mirror image study) and Pappa et al.^23^ (a 10-year mirror image study) both demonstrated that patients who discontinued PP1M experienced significantly worse outcomes in terms of both the frequency and duration of hospitalisations compared to those who remained on the treatment. These results echo our own findings, further reinforcing the advantages of sustained ALAI treatment.

### Treatment persistence

Continuation of antipsychotic medication leads to reduced relapses and hospital admissions^23^ and symptom burden^34,35^. The overall continuation rates were favourable with 53% continuing and 47% discontinuing ALAI at 5 years; these are higher than most reported in real world studies of oral and long-acting antipsychotic treatments. The retention rates for years 1 to 5 were 76.3%, 65.9%, 60.7%, 56.3% and 53.3% respectively; a decrease is seen year on year, with the greatest drop offs observed between initiation and year 1, then between years 1 and 2, after which, the year-on-year reduction in continuation rates is more gradual with few patients stopping treatment altogether in the third, fourth, and fifth year (Table 2).

As mentioned, this is the only study to report on continuation rates of ALAI over a 5-year follow-up period, though comparisons can be made with previous reports that provide data over a shorter time frame. Taylor et al.^27^ and Pantall et al.^19^ demonstrated continuation rates of 51% and 62% respectively at 1 year. If results are compared against Haloperidol, a first-generation LAI, the differences are even more stark, with only 33% remaining on treatment at 1 year^36^. This is consistent with previous findings that show improved adherence to second generation antipsychotics, despite only minor differences in symptomatic improvement^37^ and may be due to less frequent administration, alternative injection sites and improved tolerability^23^. When compared to two similar studies examining Paliperidone Palmitate 1-monthly (PP1M) over a ten year period, ALAI outperformed PP1M in the papers by Sousa Martins et al.^4^ (80%, 59%, 48% and 22% for years 2 to 5 respectively; data was not produced for those who discontinued within the first year) and, albeit to a lesser extent, by Pappa et al.^23^ (76%, 64.7%, 53.3%, 50.3% and 46.1% for years 1 to 5 respectively).

Level of treatment continuation can be considered a proxy measurement of patient preference, effectiveness and tolerability^38^. The results show that the most common reasons for discontinuation were poor compliance (24.4%) and ineffectiveness (14.1%) while poor tolerability (6.7%) was not commonly reported. This is not surprising given the low incidence of extrapyramidal side effects (EPSE), weight gain, cardiovascular abnormalities, hyperprolactinemia, hypercholesterolemia, and glucose dysregulation with Aripiprazole use^21,39–42^. Even when compared to other atypical antipsychotics, Aripiprazole displays a more favourable side-effect profile^21^. This may help to explain why poor tolerability was cited as the reason for discontinuation almost three times (17.4%) as often in a comparable study which examining PP1M over a five-year follow up period^23^.

The results show that continuation of medication is an important factor in reducing hospitalisations, while a previous study comparing ALAI and oral Aripiprazole demonstrated that those on the injectable formulation were significantly less likely to discontinue their medication^43^. Additionally, various other studies have revealed that a commonly reported reason for discontinuation is forgetfulness, both in terms of collecting medication from dispensaries as well administration of the drug itself^44,45^. Cognitive dysfunction is considered a core and common characteristic of schizophrenia^46–49^ and this could be a contributing factor to the forgetfulness around medication use. LAIs remove the need for daily medication administration and therefore may help to reduce discontinuation overall. However, patients may associate LAIs with stigma and coercion^50^, making patient choice an important factor that must always be considered^51^. Those taking medication should always be given a choice^52^, while the shared-decision making (SDM) model allows patients to make an informed choice. In SDM, clinicians and patients work collaboratively through the sharing of information, lived-experience and personal views to jointly decide on treatment; it is considered the gold standard of modern healthcare^53^ and is a widely supported tenet within the UK National Health Service (NHS)^54,55^ and further afield^56^.

### Strengths and limitations

This study was conducted independently without any external funding or support in order to minimise potential bias. It follows a design which is similar to previous studies by the authors, and others, which allows for direct comparisons with others findings. The five-year follow-up period provides an extended study duration, enhancing the applicability of the findings to real-world settings, this is especially relevant considering the chronic nature of schizophrenia and other psychotic disorders. The relatively large sample size, complemented by strong retention rates, also further enhance the validity of the results. Additionally, analysing the Schizophrenia group separately offers additional insights into the clinical application and impact of ALAI in relation to its primary treatment indication. The mirror image design allows for assessment of ‘real-world’ clinical outcomes, though the open and observational nature of the study and the absence of a control group introduce inherent limitations. As a result, the study could not account for variables such as socioeconomic factors, the aging of patients, or time-related factors, including changes in healthcare policy, service delivery models, hospitalisation criteria and clinical practices, which may have influenced outcomes independently of treatment.

An important limitation of the study was the exclusion of a large number of patients at the outset due to incomplete records and strict eligibility criteria. This may have introduced selection bias, as those lost to follow-up or lacking full records might differ in key characteristics, such as treatment adherence or illness severity. Furthermore, the study design reduces the generalisability of the results and disease severity prior to initiation was not reported on formally with the use of validated rating scales. The scope of this study also did not extend to reporting the frequency of side effects in patients who continued medication; however, such information was documented for patients who discontinued treatment.

## Conclusions

The use of ALAI has demonstrated significant real-world effectiveness by reducing both the frequency and duration of hospitalisations within this naturalistic cohort, with over half of all patients continuing treatment at 5 years follow-up. These benefits were sustained throughout the duration of the study, with few patients discontinuing treatment or requiring hospitalisation in the fourth and fifth year. The findings have important implications for the economic strain that hospital admissions impose on healthcare systems. They also reinforce the growing evidence supporting the role of ALAI, and LAIs more broadly, in promoting patient stability and improving long-term recovery outcomes. By reducing hospitalisations these treatments not only alleviate financial burden, but also enhance the quality of care and continuity for patients with chronic psychiatric conditions.

## Methods

This independent, observational, ten-year mirror-image study was conducted in the West London NHS Trust, which is a large, urban mental health provider in London, UK. Approval for the study was obtained from the department responsible for audit and naturalistic research (project number 1885), negating the need for additional formal ethical approval or written informed consent from participants.

The study cohort was formed of patients who were initiated on ALAI between 2014 and 2019 allowing for 5-year post-initiation follow up of all patients. ALAI initiation was subject to independent clinical prescribing decision and standard of care was unaffected. Inclusion criteria specified that patients must: (1) be adult patients (age > 18 years), (2) have any psychiatric diagnoses, (3) newly commenced on ALAI in this time period, and (4) possess complete hospitalisation records spanning the study period. Patients were excluded if: (1) their treatment began during a forensic admission due to typically prolonged admission periods, (2) in the event of death, and (3) patients with incomplete data during the study period e.g. because they had not been registered as being treated in our trust 5 years prior to starting ALAI or because they had were transferred from the trust in the 5 years following ALAI initiation (‘lost to follow-up’), as we were unable to establish continuation of treatment and collect their full hospitalisation data.

Data were extracted manually from electronic clinical records, encompassing demographics, primary diagnosis, substance misuse history, hospital admission frequency and duration, prior antipsychotic regimen, reasons for medication switches, discontinuation rates, and reasons for discontinuation. While disease severity was beyond the scope of this study, the number of prior admissions was used as a proxy measure.

For patients who maintained at least five years of treatment with ALAI (continuers) and those who discontinued during this timeframe (discontinuers), hospital admission rates and total bed days were evaluated and analysis was conducted using a mirror-image methodology. This compared outcomes (e.g. hospitalisations) during two equal time periods, in this case 5 years pre- and post-initiation of ALAI with each patient serving as their own control, allowing assessment of changes over time relative to treatment initiation. The mirror-image methodology is consistent with previous studies where this has been applied, and is considered a better design for evaluating long-acting treatment effectiveness or for comparing these to oral antipsychotics because it allows for more realistic comparisons of treatment outcomes in real-world settings^2,23^. These studies typically involve comparing periods of treatment with oral or other depot antipsychotics to periods of treatment with the LAI of interest within the same patients, which helps to minimize confounding factors like individual differences in illness duration and course and response to treatment. Furthermore, it has been argued that these types of studies also allow for the study of individuals not typically represented in RCTs due to the stringent inclusion and exclusion criteria and the need for a level of engagement, insight and/or concordance^2,23^.

### Statistical analysis

Descriptive statistical analysis was employed to summarise demographical, diagnostic, and clinical data, utilizing means, standard deviations (SDs), and ranges for continuous variables, while categorical variables were expressed in terms of frequencies and percentages. Discontinuation rates and primary reasons were detailed in frequency tables. Hospital length of stay was calculated using an online date difference calculator. For within-patient comparisons of inpatient admissions and duration, the nonparametric Wilcoxon signed-rank test was applied, given non-normal distribution as indicated by the Shapiro-Wilks W test; a significance threshold of 0.05 was used. Additionally, a subgroup analysis of schizophrenia-diagnosed patients was included to align with the formal indication for ALAI use in the UK and facilitate comparisons with other studies. Data analysis was conducted using SPSS Statistics for Mac (IBM, Armonk, NY, USA).

## Data Availability

The datasets generated during and analysed during the current study are not publicly available due to patient confidentiality but are available from the corresponding author on reasonable request.
